# Decrease in the number of new cancer diagnoses during the first year of the COVID-19 pandemic – cohort study of 3.5 million individuals in western Poland

**DOI:** 10.3389/fonc.2023.1230289

**Published:** 2023-12-21

**Authors:** Maciej Trojanowski, Piotr Radomyski, Witold Kycler, Irmina Maria Michalek

**Affiliations:** ^1^ Greater Poland Cancer Registry, Greater Poland Cancer Centre, Poznan, Poland; ^2^ Radiology Department, Greater Poland Cancer Centre, Poznan, Poland; ^3^ Electroradiology Department, Poznan University of Medical Sciences, Poznan, Poland; ^4^ Gastrointestinal Surgical Department, Greater Poland Cancer Centre, Poznan, Poland; ^5^ Cancer Epidemiology and Primary Prevention Department, Maria Sklodowska-Curie National Research Institute of Oncology, Warsaw, Poland

**Keywords:** cancer, cancer diagnoses, COVID-19, incidence, population-based cancer registry, SMAPE

## Abstract

**Introduction:**

The COVID-19 pandemic has considerably affected healthcare systems worldwide and is expected to influence cancer incidence, mortality, stage at diagnosis, and survival. This study aimed to assess COVID-19-related changes in cancer incidence observed in 2020 in the Greater Poland region.

**Materials and methods:**

Data from the Greater Poland Cancer Registry on cancer patients diagnosed between 2010 and 2020 were analysed. To quantify the change in the number of incident cancer cases during the COVID-19 pandemic, we calculated the standardized incidence ratio (SIR) and the incidence rate difference (IRD) to assume the pandemic-attributable gap in cancer incidence.

**Results:**

In 2020, in Greater Poland, the expected number of new cancer cases was 18 154 (9 226 among males and 8 927 among females), while the observed number was 14 770 (7 336 among males and 7 434 among females). The registered number of cancer cases decreased in 2020 by 20% (SIR 0·80, 95% CI 0·78 to 0·81) and 17% (SIR 0·83, 95% CI 0·81 to 0·85) in males and females, respectively. Among men, the most significant difference was reported for myeloma (SIR 0·59, 95% CI 0·45 to 0·77), among women for bone cancer (SIR 0·47, 95% CI 0·20 to 0·93). In females the observed incidence was higher than expected for cancer of an unspecified site (SIR 1·19, 95% CI 1·01 to 1·38). In our study, the decrease in new cancer cases was greater in males than in females.

**Discussion:**

The observed incidence was affected in most cancer sites, with the most significant deviation from the expected number in the case of myeloma. An increase in the observed incidence was reported only in women diagnosed with cancer of an unspecified site, which might reflect shortages in access to oncological diagnostics.

## 1 Introduction

The COVID-19 pandemic has considerably affected healthcare systems worldwide (1) and is expected to influence cancer epidemiological metrics, such as incidence, mortality, stage at diagnosis, and survival. The anticipated changes were caused by medical and non-medical reasons, such as limited access to healthcare services (2), fear of exposure to the virus in medical facilities, and low cancer screening participation rates.

According to literature, cancer morbidity in Europe was reduced in 2020, especially between March and May, from 22% in Sweden to 66% in Spain (1). Reports encompassing the entire 2020 period presented a lower and somewhat similar decrease in incident cancer cases compared to 2019 – 6% in Denmark (3), 6% in Sweden (4), 4% in Finland (4), and 6% in Slovenia (5). Although extensive research has been carried out on the influence of the pandemic on observed cancer incidence, no single study has compared the observed number of cases to the expected one in the Polish population, meaning that existing studies may have underestimated the influence of the pandemic by not considering the predicted year-to-year increase in cancer incidence.

The first case of COVID-19 in Poland was confirmed on the 4^th^ of March 2020. Six days later, the government implemented various restrictions, which became more severe in the following weeks. The organization of healthcare services was changed, also influencing cancer registration. Large numbers of medical staff were redirected to combat the pandemic. Hence some planned oncological surgeries were postponed or cancelled, and diagnostic tests and treatments were unavailable (6, 7). Cancer screening participation rates decreased in Poland (8–10), as in other European countries (11). Introducing telemedicine tools that received relatively positive feedback (12) partially mitigated these problems. Nevertheless, the overall impact of the pandemic on the Polish healthcare system and its performance was still considerable, as reflected by the overall number of deaths, which was approximately 15% higher than expected (13). According to Eurostat, this was one of Europe’s highest excess mortality rates (14).

This study aimed to assess COVID-19-related changes in cancer incidence observed in 2020 in the Greater Poland region (western Poland) compared with predicted values.

## 2 Materials and methods

### 2.1 Data sources, study design, and cohort selection

To assess the influence of the COVID-19 pandemic on cancer incidence in Greater Poland (Wielkopolskie), Poland, we conducted a population-based, open cohort study.

Data were obtained from the Greater Poland Cancer Registry (GPCR), an official, state-funded, Polish regional institution dealing with cancer epidemiology in Greater Poland, one of the most populous first-level administrative regions in Poland (population of 3.51 million in 2020) (15). The GPCR database covers all incident cancer cases of persons with official addresses in Greater Poland. All data are coded by qualified specialists according to the International Classification of Diseases, the tenth revision (ICD-10), and International Classification of Diseases for Oncology (ICD-O). The GPCR, as a part of the Polish Cancer Registry (PLCR), follows all the operational rules of the PLCR, comprehensively described elsewhere (16). All of the recorded cases are routinely passed through specific tools to validate the quality of the data against the recommendations of the European Network of Cancer Registries. The registration system is based on a unique Polish personal identification number (PESEL) and avoids double coding of the same patient.

Mid-year Greater Poland population estimates were obtained from Statistics Poland (15).

### 2.2 Identification of cancer cases

We identified all patients registered with a diagnosis of primary malignant neoplasms and/or *in situ* neoplasms (C00-D09, according to ICD-10) between the 1^st^ of January 2010 and the 31^st^ of December 2020. Cases diagnosed between the 1^st^ of January 2010 and the 31^st^ of December 2019 were included in the study to serve as a basis for predicting the expected number of incident cancer cases in 2020. Cases diagnosed between the 1^st^ of January 2020 and the 31^st^ of December 2020 were included to assess the influence of the COVID-19 pandemic on the number of registered cancer cases. For patients with two or more independent incident diagnoses of coexisting neoplasms, diagnoses of all primary malignant neoplasms were included in the study. We obtained the following data for all registered cases: age at diagnosis, sex, and ICD-10 code for the diagnosis. All included cancer sites were grouped and labelled, as shown in **Table S1**.

### 2.3 Statistical analyses

Crude annual incidence rate was defined as the number of new cases diagnosed per 100 000 person-years. In the denominator, we applied the mid-year population, defined as the population size on the 30^th^ of June of the respective year. Following GLOBOCAN, we performed direct age standardization for the World Standard (Segi-Doll) (17) with age-group proportions (18 age groups) to enable international comparison.

To quantify the change in the number of incident cancer cases during the COVID-19 pandemic for all cancer sites, we calculated the standardized incidence ratio (SIR), which is the ratio of the observed to expected number of new cancer cases. The observed numbers of cases were retrieved from the GPCR database. We attempted to predict the expected number of incident cancer cases in 2020, together with crude and age-standardized incidence rate (ASR), deploying GPCR data encompassing ten years before the COVID-19 pandemic (2010-2019; 159 436 individuals diagnosed with cancer), using simple linear regression analysis. A simple linear regression analysis was chosen because, according to literature, it outperforms other machine-learning models when used for predicting cancer incidence rates (18). We created 222 models (number of cases ~ year; crude incidence ~ year; standardized incidence ~ year), one for each ICD-10-sex-predicted variable combination. Formal goodness-of-fit was calculated using the symmetric mean absolute percentage error (SMAPE) (19). The SMAPE is an accuracy measure expressed as percentage errors and represents the size of a “typical” objective error in the model, overcoming the shortcomings of the original mean absolute percentage error. The 95% confidence intervals (CIs) were calculated assuming a Poisson distribution.

To assess the COVID-19 pandemic-attributable gap in cancer incidence in 2020, we calculated the incidence rate difference (IRD) by subtracting the expected ASR from the observed ASR. The IRD should be interpreted as the number of cancer cases per 100 000 person-years not identified due to the pandemic.

Statistical analyses were performed using R (version 4.1.2).

### 2.4 Compliance with ethical standards

According to Polish legislation, individual-level data from the GPCR can be used for statistics in aggregate form and for scientific purposes. The GPCR obeys strict regulations to secure confidentiality and protection of individuals. This study was conducted in accordance with the Strengthening the Reporting of Observational Studies in Epidemiology (STROBE) guidelines (20).

## 3 Results

### 3.1 Cancer incidence overall

In 2020, in Greater Poland, the expected number of new cancer cases was 18 154 (9 226 among males and 8 927 among females), while the observed number was 14 770 (7 336 among males and 7 434 among females; **Table 1**). This equates to a 20% decrease in the registered number of cancer cases in males (SIR 0.80, 95% CI 0.78 to 0.81) and 17% in females (SIR 0.83, 95% CI 0.81 to 0.85). Correspondingly, the registered ASR for all cancer sites decreased from 310 to 248 per 100 000 person-years for males and from 264 to 226 per 100 000 person-years for females. Hence, there were 62 new cancer cases less than expected per 100 000 men, and 39 new cancer cases less than expected per 100 000 women.

**Table 1:** The observed and expected crude number of new cancer cases, standardized incidence ratio (SIR) together with 95% confidence interval (CI) and symmetric mean absolute percentage error (SMAPE), crude and age-standardized incidence rate (ASR), and incidence rate difference (IRD), by sex and ICD-10 – Greater Poland, 2020.

Site (ICD-10)	Sex	Value	Cases	SIR (95% CI)	SMAPE %	Crude	ASR (World)	IRD
All sites (C00-C96, D00-D09)	Male	Observed	7 336	**0.80 (0.78 to 0.81)**	1.38	429.62	248.19	-62.24
		Expected	9 226			541.90	310.42	
	Female	Observed	7 434	**0.83 (0.81 to 0.85)**	1.28	412.23	225.81	-38.62
		Expected	8 927			495.04	264.43	
Head and neck (C00-C14)	Male	Observed	265	**0.80 (0.71 to 0.91)**	6.20	15.52	9.36	-2.41
		Expected	330			19.40	11.77	
	Female	Observed	104	**0.77 (0.63 to 0.93)**	5.35	5.77	2.87	-1.13
		Expected	135			7.54	4.00	
Digestive organs (C15-C26)	Male	Observed	1 652	**0.80 (0.76 to 0.84)**	1.86	96.75	54.59	-13.35
		Expected	2 067			121.39	67.94	
	Female	Observed	1 259	**0.84 (0.80 to 0.89)**	1.84	69.81	32.30	-5.31
		Expected	1 495			83.18	37.62	
Respiratory organs (C30-C39)	Male	Observed	1 261	**0.82 (0.77 to 0.86)**	2.84	73.85	41.19	-8.18
		Expected	1 543			90.52	49.38	
	Female	Observed	718	**0.81 (0.75 to 0.87)**	3.66	39.81	18.60	-4.93
		Expected	889			49.50	23.53	
Bones (C40-C41)	Male	Observed	12	0.67 (0.34 to 1.16)	24.49	0.70	0.61	-0.49
		Expected	18			1.08	1.10	
	Female	Observed	8	**0.47 (0.20 to 0.93)**	16.90	0.44	0.42	-0.35
		Expected	17			0.92	0.77	
Skin (C43-C44)	Male	Observed	746	**0.69 (0.64 to 0.74)**	5.39	43.69	23.51	-11.42
		Expected	1 078			63.31	34.94	
	Female	Observed	794	**0.73 (0.68 to 0.78)**	4.63	44.03	20.83	-7.08
		Expected	1 092			60.82	27.91	
Soft tissues (C45-C49)	Male	Observed	59	0.84 (0.64 to 1.09)	5.18	3.46	2.74	-0.24
		Expected	70			4.11	2.98	
	Female	Observed	58	0.84 (0.64 to 1.09)	9.55	3.22	2.01	-0.69
		Expected	69			3.82	2.70	
Breast (C50)	Male	Observed	10	0.77 (0.37 to 1.41)	21.77	0.59	0.37	-0.06
		Expected	13			0.77	0.42	
	Female	Observed	1 848	**0.88 (0.84 to 0.92)**	2.40	102.47	61.63	-6.30
		Expected	2 101			116.95	67.93	
Female genital organs (C51-C59)	Female	Observed	988	**0.83 (0.78 to 0.88)**	3.33	54.79	30.67	-5.72
		Expected	1 196			66.51	36.40	
Male genital organs (C60-C63)	Male	Observed	1 610	**0.80 (0.76 to 0.84)**	4.11	94.29	53.96	-13.24
		Expected	2 005			117.87	67.20	
Urinary tract (C64-C68)	Male	Observed	719	**0.80 (0.74 to 0.86)**	5.05	42.11	23.68	-5.79
		Expected	896			52.62	29.47	
	Female	Observed	327	**0.76 (0.68 to 0.85)**	4.10	18.13	8.99	-2.53
		Expected	431			24.02	11.53	
Central nervous system (C69-C72)	Male	Observed	154	0.92 (0.78 to 1.07)	7.06	9.02	6.40	-1.04
		Expected	168			9.89	7.44	
	Female	Observed	134	0.89 (0.74 to 1.05)	6.01	7.43	4.81	-0.65
		Expected	151			8.41	5.46	
Thyroid (C73-C75)	Male	Observed	57	**0.65 (0.49 to 0.84)**	6.85	3.34	2.46	-1.44
		Expected	88			5.19	3.90	
	Female	Observed	273	**0.73 (0.64 to 0.82)**	6.73	15.14	12.91	-3.62
		Expected	375			20.88	16.53	
Unspecified (C76-C80)	Male	Observed	172	1.06 (0.91 to 1.23)	6.61	10.07	5.59	+0.68
		Expected	162			9.51	4.91	
	Female	Observed	164	**1.19 (1.01 to 1.38)**	11.01	9.09	3.79	+0.76
		Expected	138			7.65	3.03	
Hodgkin lymphoma (C81)	Male	Observed	40	0.93 (0.66 to 1.27)	11.17	2.34	2.50	+0.24
		Expected	43			2.51	2.26	
	Female	Observed	46	1.21 (0.89 to 1.61)	7.97	2.55	2.34	+0.39
		Expected	38			2.11	1.95	
Non-Hodgkin lymphoma (C82-C88)	Male	Observed	155	**0.84 (0.71 to 0.99)**	7.41	9.08	5.99	-0.72
		Expected	184			10.79	6.70	
	Female	Observed	163	0.97 (0.83 to 1.13)	6.83	9.04	4.99	+0.12
		Expected	168			9.37	4.86	
Myeloma (C90)	Male	Observed	57	**0.59 (0.45 to 0.77)**	8.55	3.34	1.89	-1.36
		Expected	96			5.65	3.24	
	Female	Observed	64	**0.69 (0.53 to 0.88)**	16.14	3.55	1.75	-0.54
		Expected	93			5.19	2.30	
Leukaemia (C91-C96)	Male	Observed	141	**0.68 (0.58 to 0.81)**	9.93	8.26	5.91	-2.36
		Expected	206			12.09	8.27	
	Female	Observed	132	**0.79 (0.66 to 0.94)**	8.00	7.32	4.86	-0.54
		Expected	167			9.27	5.40	
In situ (D00-D09)	Male	Observed	226	**0.88 (0.77 to 1.00)**	15.08	13.24	7.45	-1.07
		Expected	258			15.20	8.51	
	Female	Observed	354	0.95 (0.86 to 1.06)	6.26	19.63	12.03	-0.49
		Expected	372			20.74	12.52	

Statistically significant values are presented in bold.

### 3.2 Cancer site and sex

The distribution of changes in registered cancer incidence was similar between sexes. Among men, the most significant difference between the observed and expected incidence was reported for myeloma (SIR 0.59, 95% CI 0.45 to 0.77; **Table 1**), followed by thyroid cancer (SIR 0.65, 95% CI 0.49 to 0.84), leukaemia (SIR 0.68, 95% CI 0.58 to 0.81), and skin cancer (SIR 0.69, 95% CI 0.64 to 0.74). Among women, the lowest significant SIR was found in bone cancer (SIR 0.47, 95% CI 0.20 to 0.93), followed by myeloma (SIR 0.69, 95% CI 0.53 to 0.88), thyroid cancer (SIR 0.73, 95% CI 0.64 to 0.82), and skin cancer (SIR 0.73, 95% CI 0.68 to 0.78). The observed incidence was higher than expected in females diagnosed with cancer of an unspecified site (SIR 1.19, 95% CI 1.01 to 1.38). There was no meaningful change in the registered incidence of tumours that are more common in younger age groups, namely soft tissues and central nervous system malignancies, and Hodgkin lymphoma.

### 3.3 Age group and sex

Among children, adolescents, and young adults, the decrease in cancer incidence was significant only among males aged 30-34 years (SIR 0.76, 95% CI 0.59 to 0.97; **Table 2**). Among older adults, the decrease in newly diagnosed cancer cases was more pronounced with rising age at diagnosis. In males all age groups ≥55 years were affected, with the most significant decrease in the observed incidence among 65-69-year-olds (SIR 0.69, 95% CI 0.66 to 0.73) and among 75-79-year-olds (SIR 0.71, 95% CI 0.66 to 0.77). Among females aged ≥55 years negative changes were also observed, with the most significant decrease in 80-84-year-olds (SIR 0.74, 95% CI 0.68 to 0.84) and 85+-year-olds (SIR 0.66, 95% CI 0.59 to 0.73).

**Table 2:** The observed and expected crude number of new cancer cases, standardized incidence ratio (SIR) together with 95% confidence interval (CI) and symmetric mean absolute percentage error (SMAPE), crude and age-standardized incidence rate (ASR), and incidence rate difference (IRD), by sex and age group – Greater Poland, 2020.

Age group	Sex	Value	Cases	SIR (95% CI)	SMAPE %	Crude	ASR (World)	IRD
0 - 4 years	Male	Observed	23	1.00 (0.63 to 1.50)	19.71	23.30	2.80	-0.11
		Expected	23			24.25	2.91	
	Female	Observed	25	1.32 (0.85 to 1.94)	14.71	26.79	3.22	+0.72
		Expected	19			20.79	2.49	
5 - 9 years	Male	Observed	11	1.00 (0.50 to 1.79)	16.05	11.00	1.10	+0.06
		Expected	11			10.40	1.04	
	Female	Observed	12	1.20 (0.62 to 2.10)	17.17	12.69	1.27	+0.26
		Expected	10			10.09	1.01	
10 - 14 years	Male	Observed	11	0.92 (0.46 to 1.64)	31.18	10.81	0.97	-0.20
		Expected	12			13.07	1.18	
	Female	Observed	9	0.75 (0.34 to 1.42)	33.37	9.43	0.85	-0.33
		Expected	12			13.12	1.18	
15 - 19 years	Male	Observed	12	0.71 (0.36 to 1.23)	12.17	13.84	1.25	-0.61
		Expected	17			20.59	1.85	
	Female	Observed	7	0.54 (0.22 to 1.11)	13.28	8.51	0.77	-0.71
		Expected	13			16.40	1.48	
20 - 24 years	Male	Observed	35	1.25 (0.87 to 1.74)	13.30	36.22	2.90	+0.50
		Expected	28			29.92	2.39	
	Female	Observed	26	0.70 (0.46 to 1.03)	8.33	27.90	2.23	-0.97
		Expected	37			39.96	3.20	
25 - 29 years	Male	Observed	47	0.85 (0.63 to 1.14)	7.21	39.69	3.18	-0.55
		Expected	55			46.56	3.72	
	Female	Observed	77	0.96 (0.76 to 1.20)	6.11	67.15	5.37	-0.12
		Expected	80			68.64	5.49	
30 - 34 years	Male	Observed	66	**0.76 (0.59 to 0.97)**	9.42	48.39	2.90	-0.63
		Expected	87			58.95	3.54	
	Female	Observed	163	1.10 (0.94 to 1.28)	4.39	123.11	7.39	+1.22
		Expected	148			102.79	6.17	
35 - 39 years	Male	Observed	107	0.95 (0.78 to 1.14)	6.78	70.42	4.23	-0.16
		Expected	113			73.04	4.38	
	Female	Observed	239	1.00 (0.88 to 1.14)	5.81	161.18	9.67	+0.13
		Expected	239			158.94	9.54	
40 - 44 years	Male	Observed	146	0.96 (0.81 to 1.13)	7.26	102.04	6.12	-0.22
		Expected	152			105.77	6.35	
	Female	Observed	348	**0.90 (0.81 to 1.00)**	3.62	248.95	14.94	-1.81
		Expected	388			279.10	16.75	
45 - 49 years	Male	Observed	181	**0.87 (0.74 to 1.00)**	4.93	148.49	8.91	-2.25
		Expected	209			185.95	11.16	
	Female	Observed	414	0.92 (0.83 to 1.01)	5.08	342.85	20.57	-3.84
		Expected	450			406.79	24.41	
50 - 54 years	Male	Observed	312	1.01 (0.90 to 1.12)	3.32	311.59	15.58	-1.53
		Expected	310			342.11	17.11	
	Female	Observed	484	0.94 (0.86 to 1.03)	2.62	474.92	23.75	-3.78
		Expected	515			550.44	27.52	
55 - 59 years	Male	Observed	591	**0.80 (0.74 to 0.87)**	3.39	592.66	23.71	-3.93
		Expected	739			691.01	27.64	
	Female	Observed	653	**0.81 (0.75 to 0.87)**	3.43	615.63	24.63	-3.82
		Expected	808			711.05	28.44	
60 - 64 years	Male	Observed	1 114	**0.74 (0.70 to 0.79)**	2.60	1005.30	40.21	-11.48
		Expected	1 499			1292.38	51.70	
	Female	Observed	1 012	**0.80 (0.76 to 0.85)**	2.54	810.23	32.41	-5.86
		Expected	1 259			956.77	38.27	
65 - 69 years	Male	Observed	1 500	**0.69 (0.66 to 0.73)**	6.22	1558.15	46.74	-13.38
		Expected	2 169			2004.18	60.13	
	Female	Observed	1 273	**0.75 (0.71 to 0.79)**	7.46	1075.41	32.26	-6.29
		Expected	1 706			1285.12	38.55	
70 - 74 years	Male	Observed	1 585	**0.96 (0.91 to 1.00)**	9.63	2163.29	43.27	-13.82
		Expected	1 658			2854.27	57.09	
	Female	Observed	1 219	1.02 (0.97 to 1.08)	9.46	1230.37	24.61	-6.26
		Expected	1 193			1543.49	30.87	
75 - 79 years	Male	Observed	715	**0.71 (0.66 to 0.77)**	2.71	2309.43	23.09	-8.03
		Expected	1 002			3112.04	31.12	
	Female	Observed	583	**0.74 (0.68 to 0.80)**	4.07	1199.64	12.00	-3.25
		Expected	788			1524.88	15.25	
80 - 84 years	Male	Observed	545	**0.78 (0.72 to 0.85)**	3.87	2336.25	11.68	-2.90
		Expected	697			2916.85	14.58	
	Female	Observed	513	**0.74 (0.68 to 0.81)**	2.77	1133.78	5.67	-1.84
		Expected	690			1502.53	7.51	
85+ years	Male	Observed	335	**0.75 (0.68 to 0.84)**	7.62	1911.66	9.56	-2.99
		Expected	444			2509.36	12.55	
	Female	Observed	377	**0.66 (0.59 to 0.73)**	5.35	845.10	4.23	-2.07
		Expected	574			1259.98	6.30	

Statistically significant values are presented in bold.

## 4 Discussion

### 4.1 The relevance of the study

We assessed the influence of the COVID-19 pandemic on cancer incidence in Greater Poland, one of the largest administrative regions in Poland. In contrast with existing literature which compares yearly epidemiological observations, this study uses a calculation method juxtaposing observed new cancer cases with expected numbers. The added value of this study lies in a more precise assessment of the gap caused by the COVID-19 pandemic, as 2020 data are contrasted with predicted numbers for this year rather than past epidemiological data, taking into account expected changes in cancer incidence over time.

In addition, contrary to other European cancer registries that reported disruptions in cancer registration (21), during the first wave of the COVID-19 pandemic, the GPCR continued the registration process and provided a reliable dataset for such an analysis. The continued work of the GPCR was possible because of the hybrid work model and the division of the registry staff into separate teams without close contact.

### 4.2 Main findings of the study in the context of Polish literature

One of our main observations was the decrease in the registered number of new cancer cases in 2020 by 20% in males and 17% in females compared with the number of cases expected in that year. Our findings broadly support previous estimates by the PLCR, which compared the number of incident cancer cases in 2020 versus 2019 and reported a difference of -15% in Polish males and -11% in Polish females (22). The differences described by the PLCR varied by region and were highest in one of the northern regions, Pomorskie (-30%). For Greater Poland, the reported difference was -15% in males and -11% in females. The discrepancy between the present analysis and the PLCR report relates to the fact that this study compared the 2020 observations with expected numbers derived from a broader historical period, which reflects a further increase in cancer incidence more accurately. In other words, we compared the observed number of cases with a broader data set, encompassing 2019 data.

Other Polish observations, which were based on multi-disciplinary team (MDT) reports, showed a 20% drop in new, suspected cancer cases in 2020 compared to 2019 (23). One of the possible explanations for such large differences in MDT reports (percentage difference greater than in our study) might be the fact that not all cases analysed by the MDT had to be later confirmed as malignant neoplasms.

### 4.3 Main findings of the study in the context of European literature

The first published reports on the impact of the COVID-19 pandemic on cancer incidence metrics deployed year-over-year comparisons. They showed that there were fewer incident cancer cases in 2020 than in 2019. Notwithstanding the differences in the calculation method and the periods analysed, one can observe that the decrease in the number of new cancer cases found in our study is larger than that in other European countries. For example, a Belgian report showed that despite a 44% reduction in new cancer diagnoses in April 2020 compared with April 2019, the annual decline in 2020 reached 6% (24). Contrary to our study, the most significant decrease was observed in head and neck cancers. On the other hand, our observations were similar regarding childhood cancers (no relative change found) and the impact of age on the decrease in cancer incidence. However, while in Poland we observed noteworthy changes in patients aged ≥55 years, in Belgium considerable differences were observed in individuals aged 80 years or older. Another example is a German study, which compared the number of patients newly diagnosed with cancer in general and specialised medical practices between April 2020 and March 2021 with the corresponding numbers from April 2019 to March 2020, and found a 7% decrease in adults (25). A Dutch study also found a considerable change in the number of new cancer diagnoses for all cancer sites during the first months of the pandemic, with the biggest difference in the case of skin and breast cancers (26). The authors indicated problems with access to GPs when displaying non-specific symptoms, choosing telemedicine services for most non-acute issues, and postponing hospital-based diagnostic examinations as the main possible reasons for such observations. In Poland, these factors may have also contributed to the decrease in the observed number of incident cancer cases (12).

### 4.4 Cancer incidence gap by sex and age

In our study, the decrease in new cancer cases was greater in males than in females. This could be linked to higher COVID-19 mortality observed among men especially in urban areas (27), which might have influenced the number of malignancies potentially undiagnosed due to COVID-19-related deaths. Furthermore, the impact of the pandemic on the observed cancer incidence varied by age group and was most significant in patients aged ≥55 years. These observations are somewhat coherent with those from Germany (25) and the Spanish region of Catalonia (28). They might relate to the fact that stay-at-home advice was addressed mainly to seniors. Contrary to an English study, which reported a decrease in new childhood cancer diagnoses (29), neither our analysis nor Canadian (30) or German (31) reports confirmed such a trend.

### 4.5 Changes in incidence by cancer site

*Head and neck.* Consistently with previous data from the USA (-25%) (32) and Belgium (-14%) (24), we observed a 20% reduction in new head and neck cancer diagnoses among males and 23% among females.

*Digestive system.* The COVID-19 pandemic posed severe challenges to all aspects of colorectal cancer diagnostics and treatment in many settings (33, 34). Such limited access to diagnosis and treatment in Greater Poland was reflected by the decrease in the number of new digestive system cancers in 2020 by 20% in males and 16% in females. A smaller decline in incidence was reported in Denmark (10%) (3) and Belgium (11%) (24), whereas a larger decrease was found in Hungary (20%) (35) and Spain (39%) (36). Since it has been proven that a four month delay in colorectal cancer treatment results in higher fatality rates and lower survival (37), further analyses need to assess the impact of the pandemic on these metrics.

*Respiratory system.* Despite eminence-based expectations that numerous computed tomography scans of the chest performed during the pandemic would increase the number of incident lung cancers, in 2020, we observed almost 20% fewer new respiratory cancer cases than expected. Other Dutch and Indian studies have also rejected the hypothesis of a higher number of incidental diagnoses of lung cancer during the pandemic (38, 39). The Polish incidence gap for respiratory system cancers was considerably larger than in other European countries, including Hungary (-14%) (35), Denmark (-4%) (3), and Belgium (-2%) (24). Considering the above and the fact that Polish COVID-19-positive patients diagnosed with lung cancer were characterised by worse performance upon admission, more advanced stage at diagnosis, and more deaths before treatment during the pandemic (40), further survival analyses should be performed.

*Breast.* In Greater Poland, in 2020, the number of new female breast cancer cases was 12% lower than expected, which is consistent with observations from other former Eastern Bloc countries, including Hungary (-16%) (35) and Slovenia (-17%) (5). A smaller incidence gap was observed in Western Europe, namely Belgium (6%) (24) and Denmark (8%) (3). Although analyses of patients diagnosed in 2020 have found no differences in stage at diagnosis (41), the delay in breast cancer diagnosis is expected to affect the stage in the years following the pandemic. Such signals came from Greater Poland, where the first significant breast cancer stage shift was observed in patients diagnosed in 2021 (42).

*Female genital organs.* Our findings show a 17% reduction in the number of registered cancer cases. Even higher decreases were reported in 2020 in Austria (45%) (43) and Northern England (26%) (44). The group of female genital malignancies is diverse, according to the average age at diagnosis and the presence of population-based screening. Hence, the overall COVID-19 influence on the number of observed diagnoses should only serve as an introduction for further studies.

*In situ neoplasms.* The ability to diagnose cancer at an early stage is one of the measures of healthcare system oncological diagnostics performance. In Greater Poland, we identified a 12% decrease in male *in situ* cancers and no significant change in females. This is probably caused by the structure of preinvasive malignancies that differs between the sexes. In men, it is dominated by bladder cancer and in women by breast cancer. The findings regarding *in situ* neoplasms correspond with invasive tumours of the male urinary tract (-20%) and female breast (-12%).

The key strength of this study is the usage of the population-based cancer registry data, covering the whole population of Greater Poland with high completeness, achieved by active registration practices. Another advantage of this study is providing a comparison with the predicted values based on a long history of the registry operation using well-fitted models with acceptable SMAPE. The study is somewhat limited by the lack of a more detailed picture of pandemic-related changes in cancer burden in 2021, which is caused by lag time in cancer registration, typical for cancer registries.

The COVID-19 pandemic negatively affected the registered cancer incidence in Greater Poland in 2020. The number of new cancer diagnoses decreased by 20% among males and 17% among females, more than among other European populations. The gap was most significant in the 55+-year age group. The observed decrease could be linked to the shortages in Polish healthcare resources, already limited in the pre-pandemic period, which struggled to maintain oncological services, especially outside of the comprehensive cancer centres and due to stay-at-home policy primarily enforced among seniors.

The observed incidence was affected in most cancer sites, with the most significant deviation from the expected one in the case of myeloma. An increase in the observed incidence was reported only in women diagnosed with cancer of an unspecified site, which might reflect shortages in access to oncological diagnostics.

The reported decrease in cancer incidence due to the pandemic indicates the need for further studies focused on the possible consequences of postponed diagnostics and treatment, including changes in survival metrics and healthcare expenditures.

## Data availability statement

The agregated data supporting the conclusions of this article will be made available by the authors, without undue reservation.

## Author contributions

Conceptualization: MT, IM, PR, WK. Methodology: IM, MT. Software: IM. Formal analysis: IM. Data Curation: MT, IM. Project administration: MT. Writing – original draft: MT, IM. Writing – review & editing: MT, IM, PR, WK. All authors contributed to the article and approved the submitted version.

## References

[1] OECD, European Union. Health at a glance: europe 2020: state of health in the EU cycle. OECD (2020). doi: 10.1787/82129230-en

[2] Angelini M Teglia F Astolfi L Casolari G Boffetta P. Decrease of cancer diagnosis during COVID-19 pandemic: a systematic review and meta-analysis. Eur J Epidemiol (2023) 38:31–8. doi: 10.1007/s10654-022-00946-6 PMC9807424 36593334

[3] Skovlund CW Friis S Christensen J Nilbert MC Mørch LS. Drop in cancer diagnosis during the COVID-19 pandemic in Denmark: assessment of impact during 2020. Acta Oncol (2022) 61:658–61. doi: 10.1080/0284186X.2021.2024879 35020549

[4] Johansson ALV Larønningen S Skovlund CW Kristiansen MF Mørch LS Friis S. The impact of the COVID -19 pandemic on cancer diagnosis based on pathology notifications: A comparison across the Nordic countries during 2020. Intl J Cancer (2022) 151:381–95. doi: 10.1002/ijc.34029 PMC9087674 35419824

[5] Zagar T Tomsic S Zadnik V Bric N Birk M Vurzer B. Impact of the COVID-19 epidemic on cancer burden and cancer care in Slovenia: a follow-up study. Radiol Oncol (2022) 56:488–500. doi: 10.2478/raon-2022-0050 36503711 PMC9784361

[6] Stefura T Rymarowicz J Wysocki M Szeliga J Wallner G Pędziwiatr M. Surgical care in Poland after COVID-19 outbreak: a national survey. Folia Med Cracov (2020) 60:33–51. doi: 10.24425/fmc.2020.135794 33582744

[7] Malicki J Martenka P Dyzmann-Sroka A Paczkowska K Leporowska E Suchorska W. Impact of COVID-19 on the performance of a radiation oncology department at a major comprehensive cancer centre in Poland during the first ten weeks of the epidemic. Rep Pract Oncol Radiother (2020) 25:820–7. doi: 10.1016/j.rpor.2020.08.001 PMC7429079 32837336

[8] Koczkodaj P Kamiński M Ciuba A Didkowska J. Cancer screening coverage in Poland – from bad to better to the worst during the SARS-CoV-2 pandemic. Arch Med Sci (2021) 17:1132–3. doi: 10.5114/aoms/134239 PMC8314399 34336043

[9] Koczkodaj P Sulkowska U Kamiński MF Didkowska J. SARS-CoV-2 as a new possible long-lasting determining factor impacting cancer death numbers. Based on the example of breast, colorectal and cervical cancer in Poland. Nowotwory J Oncol (2021) 71:42–6. doi: 10.5603/NJO.2021.0007

[10] Appel S Lawrence YR Symon Z Kaidar-Person O. COVID-RO study: the radiation oncology practice at times of COVID-19 outbreak - international survey. Rep Pract Oncol Radiother (2021) 26:20–8. doi: 10.5603/RPOR.a2021.0003 PMC8086710 33948298

[11] Dinmohamed AG Cellamare M Visser O de Munck L Elferink MAG Westenend PJ. The impact of the temporary suspension of national cancer screening programmes due to the COVID-19 epidemic on the diagnosis of breast and colorectal cancer in the Netherlands. J Hematol Oncol (2020) 13:147. doi: 10.1186/s13045-020-00984-1 33148289 PMC7609826

[12] Mularczyk-Tomczewska P Zarnowski A Gujski M Jankowski M Bojar I Wdowiak A. Barriers to accessing health services during the COVID-19 pandemic in Poland: A nationwide cross-sectional survey among 109,928 adults in Poland. Front Public Health (2022) 10:986996. doi: 10.3389/fpubh.2022.986996 36159267 PMC9495711

[13] Pikala M Burzyńska M. Excess mortality during the coronavirus pandemic (COVID-19) in Poland. Eur J Public Health (2022) 32:ckac131.197. doi: 10.1093/eurpub/ckac131.197

[14] Excess mortality in the EU between January 2020 and December 2022. Eurostat . Available at: https://ec.europa.eu/eurostat/statistics-explained/index.php?title=Excess_mortality_-_statistics#Excess_mortality_in_the_EU_between_January_2020_and_December_2022 (Accessed February 23, 2023).

[15] Główny Urząd Statystyczny / Obszary tematyczne / Ludność . Available at: https://stat.gov.pl/obszary-tematyczne/ludnosc/ (Accessed March 2, 2023).

[16] Didkowska J Wojciechowska U Michalek IM Caetano dos Santos FL. Cancer incidence and mortality in Poland in 2019. Sci Rep (2022) 12:10875. doi: 10.1038/s41598-022-14779-6 35760845 PMC9237124

[17] Bray F Ferlay J. Chapter 7: Age standardization IARC scientific publications (2014) 164 Pt 1 (2014):112–5. 27220130

[18] Sekeroglu B Tuncal K. Prediction of cancer incidence rates for the European continent using machine learning models. Health Inf J (2021) 27:146045822098387. doi: 10.1177/1460458220983878 33506703

[19] Flores BE. A pragmatic view of accuracy measurement in forecasting. Omega (1986) 14:93–8. doi: 10.1016/0305-0483(86)90013-7

[20] von Elm E Altman DG Egger M Pocock SJ Gøtzsche PC Vandenbroucke JP. Strengthening the reporting of observational studies in epidemiology (STROBE) statement: guidelines for reporting observational studies. BMJ (2007) 335:806–8. doi: 10.1136/bmj.39335.541782.AD PMC2034723 17947786

[21] Neamţiu L Martos C Giusti F Negrão Carvalho R Randi G Dimitrova N. Impact of the first wave of the COVID-19 pandemic on cancer registration and cancer care: a European survey. Eur J Public Health (2022) 32:311–5. doi: 10.1093/eurpub/ckab214 PMC8975538 34935934

[22] Wojciechowska U. Cancer in Poland in 2020 . Available at: https://onkologia.org.pl/sites/default/files/publications/2023-01/nowotwory_2020.pdf (Accessed March 12, 2023).

[23] Maluchnik M Podwójcic K Więckowska B. Decreasing access to cancer diagnosis and treatment during the COVID-19 pandemic in Poland. Acta Oncol (2021) 60:28–31. doi: 10.1080/0284186X.2020.1837392 33104414

[24] Peacock HM Tambuyzer T Verdoodt F Calay F Poirel HA De Schutter H. Decline and incomplete recovery in cancer diagnoses during the COVID-19 pandemic in Belgium: a year-long, population-level analysis. ESMO Open (2021) 6:100197. doi: 10.1016/j.esmoop.2021.100197 34474811 PMC8411068

[25] Jacob L Kalder M Kostev K. Decrease in the number of patients diagnosed with cancer during the COVID-19 pandemic in Germany. J Cancer Res Clin Oncol (2022) 148:3117–23. doi: 10.1007/s00432-022-03922-5 PMC8764247 35041059

[26] Dinmohamed AG Visser O Verhoeven RHA Louwman MWJ van Nederveen FH Willems SM. Fewer cancer diagnoses during the COVID-19 epidemic in the Netherlands. Lancet Oncol (2020) 21:750–1. doi: 10.1016/S1470-2045(20)30265-5 PMC7252180 32359403

[27] Kuropka I Rossa A Wróblewska W Wojtyniak B Śleszyński P. Pandemia i jej skutki zdrowotne i demograficzne. Pandemia i jej skutki zdrowotne i demograficzne (2021). doi: 10.24425/140474

[28] Ribes J Pareja L Sanz X Mosteiro S Escribà JM Esteban L. Cancer diagnosis in Catalonia (Spain) after two years of COVID-19 pandemic: an incomplete recovery. ESMO Open (2022) 7:100486. doi: 10.1016/j.esmoop.2022.100486 35714476 PMC9197337

[29] Saatci D Oke J Harnden A Hippisley-Cox J. Childhood, teenage and young adult cancer diagnosis during the first wave of the COVID-19 pandemic: a population-based observational cohort study in England. Arch Dis Child (2022) 107:740–6. doi: 10.1136/archdischild-2021-322644 35318196

[30] Pelland-Marcotte M-C Xie L Barber R Elkhalifa S Frechette M Kaur J. Incidence of childhood cancer in Canada during the COVID-19 pandemic. CMAJ (2021) 193:E1798–806. doi: 10.1503/cmaj.210659 PMC8654886 34844937

[31] Erdmann F Wellbrock M Trübenbach C Spix C Schrappe M Schüz J. Impact of the COVID-19 pandemic on incidence, time of diagnosis and delivery of healthcare among paediatric oncology patients in Germany in 2020: Evidence from the German Childhood Cancer Registry and a qualitative survey. Lancet Reg Health Eur (2021) 9:100188. doi: 10.1016/j.lanepe.2021.100188 34514453 PMC8423844

[32] Kiong KL Diaz EM Gross ND Diaz EM Hanna EY. The impact of COVID -19 on head and neck cancer diagnosis and disease extent. Head Neck (2021) 43:1890–7. doi: 10.1002/hed.26665 PMC8013528 33650276

[33] Mazidimoradi A Hadavandsiri F Momenimovahed Z Salehiniya H. Impact of the COVID-19 pandemic on colorectal cancer diagnosis and treatment: a systematic review. J Gastrointest Canc (2021) 54,1(2023):171–87. doi: 10.1007/s12029-021-00752-5 PMC8628028 34843058

[34] Morris EJA Goldacre R Spata E Mafham M Finan PJ Shelton J. Impact of the COVID-19 pandemic on the detection and management of colorectal cancer in England: a population-based study. Lancet Gastroenterol Hepatol (2021) 6:199–208. doi: 10.1016/S2468-1253(21)00005-4 33453763 PMC7808901

[35] Elek P Csanádi M Fadgyas-Freyler P Gervai N Oross-Bécsi R Szécsényi-Nagy B. Heterogeneous impact of the COVID-19 pandemic on lung, colorectal and breast cancer incidence in Hungary: results from time series and panel data models. BMJ Open (2022) 12:e061941. doi: 10.1136/bmjopen-2022-061941 PMC9393855 35981776

[36] Hijos-Mallada G Alfaro E Navarro M Cañamares P Ariño I Charro M. Impact of the COVID-19 pandemic in colorectal cancer diagnosis and presentation. Gastroenterología y Hepatología (2023) 46,9(2023):702–9. doi: 10.1016/j.gastrohep.2023.01.007 PMC9882881 36716926

[37] Luo Q O’Connell DL Yu XQ Kahn C Caruana M Pesola F. Cancer incidence and mortality in Australia from 2020 to 2044 and an exploratory analysis of the potential effect of treatment delays during the COVID-19 pandemic: a statistical modelling study. Lancet Public Health (2022) 7:e537–48. doi: 10.1016/S2468-2667(22)00090-1 PMC9159737 35660215

[38] Kilsdonk ID Roos MP de Bresser P Reesink HJ Peringa J. Frequency and spectrum of incidental findings when using chest CT as a primary triage tool for COVID-19. Eur J Radiol Open (2021) 8(2021):100366. doi: 10.1016/j.ejro.2021.100366 34189189 PMC8226060

[39] Dündar İ Özkaçmaz S Durmaz F Çoban LT Aygün G Yıldız R. Detection of incidental findings on chest CT scans in patients with suspected COVID-19 pneumonia. Eastern J Med (2021) 26:566–74. doi: 10.5505/ejm.2021.26428

[40] Mojsak D Dębczyński M Kuklińska B Minarowski Ł Kasiukiewicz A Moniuszko-Malinowska A. Impact of COVID-19 in patients with lung cancer: A descriptive analysis. IJERPH (2023) 20:1583. doi: 10.3390/ijerph20021583 36674340 PMC9866646

[41] Tonneson JE Hoskin TL Day CN Durgan DM Dilaveri CA Boughey JC. Impact of the COVID-19 pandemic on breast cancer stage at diagnosis, presentation, and patient management. Ann Surg Oncol (2022) 29:2231–9. doi: 10.1245/s10434-021-11088-6 PMC8609838 34812981

[42] Trojanowski M Radomyski P Matuszewski K Litwiniuk M Wierzchosławska E Kycler W. Impact of the COVID-19 pandemic on breast cancer stage at diagnosis in a regional cancer center in Poland between 2019 and 2021. J Pers Med (2022) 12:1486. doi: 10.3390/jpm12091486 36143271 PMC9503146

[43] Knoll K Reiser E Leitner K Kögl J Ebner C Marth C. The impact of COVID-19 pandemic on the rate of newly diagnosed gynecological and breast cancers: a tertiary center perspective. Arch Gynecol Obstet (2022) 305:945–53. doi: 10.1007/s00404-021-06259-5 PMC8460407 34559295

[44] Davies JM Spencer A Macdonald S Dobson L Haydock E Burton H. Cervical cancer and COVID-an assessment of the initial effect of the pandemic and subsequent projection of impact for women in England: A cohort study. BJOG (2022) 129:1133–9. doi: 10.1111/1471-0528.17098 PMC9303941 35015334

