# Supplementary material for: Decrease in the number of new cancer diagnoses during the first year of the COVID-19 pandemic – cohort study of 3.5 million individuals in western Poland
Source: Front Oncol. 2023 Dec 21;13:1230289. doi: 10.3389/fonc.2023.1230289 (PMC10765942; doi:10.3389/fonc.2023.1230289)
Supplement: Supplementary file 1 [file Table_1.docx]

**Table S1.** ICD-10 codes and cancer sites included in the analysis, together with respective collective names (labels).

| **ICD-10** | **Sites** | **Label** |
| --- | --- | --- |
| C00-C14 | Lip, oral cavity, and pharynx | Head and neck |
| C15-C26 | Digestive organs | Digestive organs |
| C30-C39 | Respiratory and intrathoracic organs | Respiratory organs |
| C40-C41 | Bone and articular cartilage | Bones |
| C43-C44 | Melanoma and other skin cancers | Skin |
| C45-C49 | Mesothelial and soft tissue | Soft tissues |
| C50 | Breast | Breast |
| C51-C58 | Female genital organs | Female genital organs |
| C60-C63 | Male genital organs | Male genital organs |
| C64-C68 | Urinary tract | Urinary tract |
| C69-C72 | Eye, brain, and other parts of central nervous system | Central nervous system |
| C73-C75 | Thyroid and other endocrine glands | Thyroid |
| C76-C80 | Ill-defined, secondary, and unspecified sites | Unspecified |
| C81 | Hodgkin lymphoma | Hodgkin lymphoma |
| C82-C88 | Non-Hodgkin lymphoma | Non-Hodgkin lymphoma |
| C90 | Multiple myeloma and malignant plasma cell neoplasms | Myeloma |
| C91-C96 | Leukaemia | Leukaemia |
| C97 | Independent (primary) multiple sites | Multiple |
| D01-D09 | In situ neoplasms | In situ |
